# Artificial Intelligence in Cerebrovascular Imaging: A Targeted Review of Aneurysm Detection and Rupture Risk Prediction

**DOI:** 10.31662/jmaj.2025-0327

**Published:** 2025-12-12

**Authors:** Duong Le

**Affiliations:** 1 Department of Biomedical Engineering, University of Massachusetts Amherst, Amherst, MA, USA

**Keywords:** artificial intelligence, machine learning, deep learning, radiomics, cerebral aneurysm, rupture risk prediction, medical imaging, neural networks

## Abstract

Cerebral aneurysms are a potentially life-threatening vascular pathology that can lead to subarachnoid hemorrhage, a neurological emergency associated with high morbidity and mortality. Traditional imaging-based assessments (largely centered on aneurysm size, shape, and location) often fall short in accurately predicting rupture risk. This limitation highlights the need for more advanced, individualized diagnostic strategies. Recent advancements in artificial intelligence (AI) have introduced powerful tools capable of transforming cerebrovascular imaging and aneurysm management. This narrative review synthesizes published studies on the application of AI in cerebrovascular imaging, focusing on its potential to aid in aneurysm detection and rupture risk prediction. It examines the evolving role of AI through three primary technological approaches: radiomics, machine learning (ML), and deep learning (DL). Radiomics enables the extraction of quantitative features from imaging data, revealing patterns and morphological indicators that may not be visible to the human eye. ML models synthesize imaging, clinical, and hemodynamic data to predict rupture risk with greater precision than traditional scoring tools. DL techniques, particularly convolutional neural networks, automate aneurysm detection and interpretation directly from raw image data.

What sets this review apart from previous literature is its integrative approach: rather than focusing narrowly on one AI technique or imaging modality, it unifies radiomics, ML, and DL under a single framework and evaluates their clinical applications across both detection and risk prediction. Furthermore, it emphasizes emerging solutions like hybrid modeling, explainable AI, and multimodal data fusion, which are critical for real-world clinical translation. However, current AI-based methods remain at the investigational stage and have not yet been validated clinically, experimentally, or against existing diagnostic standards. Importantly, this review situates AI methods relative to established clinical benchmarks, including radiologist interpretation and risk scores such as PHASES (Population, Hypertension, Age, Size of aneurysm, Earlier subarachnoid, Hypertension, Age, Size of aneurysm, Earlier subarachnoid hemorrhage, and Site of aneurysm) and hemorrhage, and Site of aneurysm) and ELAPSS ( (Earlier subarachnoid hemorrhage Earlier subarachnoid hemorrhage, Location of aneurysm, Age, Population, Size of aneurysm, and Shape of aneurysm), Location of aneurysm, Age, Population, Size of aneurysm, and Shape of aneurysm), and emphasizes that rigorous prospective validation is essential before widespread adoption. It also proposes practical implementation strategies, including decision support integration, standardization protocols, and federated learning to enable secure data collaboration. By addressing both technical innovation and translational challenges, this review offers a clinician-focused roadmap that advances the field beyond theoretical models toward personalized aneurysm care. In doing so, it aims to reduce rupture rates and improve patient outcomes through precision medicine powered by AI.

## Introduction

Cerebral aneurysms are abnormal dilations of intracranial arteries, often occurring at high-flow bifurcation points such as the anterior communicating artery, middle cerebral artery, or internal carotid artery ^(1)^. Affecting 2%-5% of the population, they typically remain asymptomatic until rupture, which results in subarachnoid hemorrhage, a life-threatening condition characterized by sudden severe headache, loss of consciousness, and high morbidity and mortality―nearly 50% of patients die, and survivors often face permanent neurological deficits ^(2), (3)^. Early detection and accurate risk assessment are therefore essential. Imaging modalities such as computed tomography angiography (CTA), magnetic resonance angiography (MRA), and digital subtraction angiography are routinely used to evaluate aneurysm morphology, but interpretation may vary with radiologist expertise, particularly for small or complex aneurysms ^(4), (5)^. Clinical scores such as PHASES, which estimate rupture risk based on aneurysm size, location, and patient demographics, provide population-level guidance but lack precision for individual patients, as some small aneurysms rupture while larger lesions remain stable ^(6), (7)^.

Aneurysm progression is driven by complex biomechanical and biological processes, including hemodynamic stress, vascular inflammation, extracellular matrix remodeling, and genetic predisposition ^(8), (9)^. Translating these diverse factors into clinical decision-making remains difficult due to the complexity of data and the absence of standardized predictive tools. Artificial intelligence (AI), particularly machine learning (ML) and deep learning (DL), has emerged as a potential solution by identifying nonlinear patterns across large multimodal datasets, including imaging, clinical variables, and computational hemodynamics. Convolutional neural networks (CNNs), for example, have demonstrated near-human performance in lesion detection across a range of imaging tasks ^(10), (11), (12)^. However, applications of AI to aneurysm imaging face important challenges, including limited dataset sizes, restricted generalizability, interpretability, and ethical concerns, with many studies lacking standardized methodology, benchmark comparisons, or external validation ^(13)^.

This manuscript is a narrative review synthesizing published studies on AI in cerebrovascular imaging, not a protocol or original dataset. We focus on two major applications: aneurysm detection and rupture risk prediction. Importantly, we situate these methods relative to established clinical benchmarks, including radiologist interpretation of CTA and MRA and widely used risk models such as PHASES and ELAPSS, which remain the standards of comparison for clinical decision-making. The goal is not to claim superiority over current benchmarks but to outline the pathway by which AI could complement existing practices and ultimately advance personalized, proactive aneurysm care.

## Overview of AI Techniques in Medical Imaging

### Radiomics: Quantitative image analysis

Radiomics converts medical images into detailed numerical datasets by extracting quantitative features that describe aneurysm characteristics, offering insights beyond what visual inspection can achieve. This approach quantifies shape, texture, and intensity variations, providing a level of detail that can indicate rupture risk with precision unattainable by human observation alone ^(14)^. The radiomics pipeline begins with image acquisition and preprocessing. High-resolution images from CTA or MRA are standardized to ensure consistency across different scanners and protocols. This involves adjusting image brightness through intensity normalization, reducing noise to enhance clarity, and resampling voxels to uniform sizes ^(15)^. Traditionally, radiologists relied on visual inspection, manually measuring basic metrics like aneurysm diameter, a process that was subjective and prone to variability due to differences in expertise or image quality ^(16)^. Radiomics automates and expands this process, reducing human error and systematically capturing hundreds of features.

Segmentation follows, where the aneurysm is isolated within the 3-dimensional (3D) image by defining its boundaries. This can be done manually by radiologists, through semi-automatic software using edge-detection algorithms, or via fully automatic methods like thresholding. Accurate segmentation is critical, as errors can distort subsequent analyses, much like misaligning a microscope’s focus ^(17)^. Traditional manual segmentation was time-consuming and susceptible to inter-observer variability, often missing subtle morphological details. Radiomics employs automated or semi-automated tools to enhance precision and consistency, significantly improving efficiency ^(18)^.

Feature extraction comes next, where algorithms compute a wide range of quantitative descriptors. These include shape metrics like volume and sphericity, which describe the aneurysm’s 3D geometry; intensity metrics such as mean brightness, which reflect tissue properties; and texture metrics that capture surface patterns and internal structural variations potentially indicative of wall instability ^(19)^. Wavelet transforms further analyze the image at multiple scales, revealing structural details invisible to traditional visual assessments ^(20)^. In contrast, traditional approaches relied on simplistic metrics like maximum diameter, which failed to capture complex morphological or textural cues, limiting their predictive power ^(21)^.

To manage the large number of extracted features, feature selection identifies the most predictive ones, preventing models from memorizing data rather than learning patterns. Techniques like LASSO regression or mutual information analysis filter out redundant features, akin to selecting the most relevant evidence for a case ^(14)^. These selected features are then integrated into ML models, such as logistic regression, to predict outcomes like rupture risk. These models are trained on labeled datasets and validated through cross-validation to ensure reliability ^(15)^. Unlike traditional methods that used basic size thresholds (e.g., >7 mm indicating high risk) with limited accuracy, radiomics-based models achieve superior performance by incorporating diverse, quantitative features, enabling more nuanced risk assessments ^(20)^. The interpretability of radiomics features, such as surface irregularity linked to biomechanical stress, aligns with clinical reasoning, making it valuable for hypothesis generation and adoption in aneurysm care ^(18)^.

### ML: Pattern recognition with structured data

ML enables computers to identify patterns in structured data without explicit programming, synthesizing diverse inputs to predict aneurysm behavior. This approach integrates radiomic features, clinical variables, and hemodynamic parameters to provide robust risk predictions, functioning like a skilled analyst piecing together evidence ^(4)^. The process starts with data collection from multiple sources, including radiomic features from imaging, clinical data like age or hypertension from medical records, and hemodynamic metrics such as wall shear stress from computational fluid dynamics simulations ^(22)^. Missing data, common in clinical settings, is addressed through imputation techniques, such as estimating values based on similar patients, ensuring comprehensive analysis ^(23)^. Traditionally, clinicians relied on manual chart reviews and basic statistical models, which were labor-intensive and limited by incomplete data or simplistic assumptions, often missing complex interactions ^(24)^. ML automates data integration and handles missing values systematically, improving efficiency and accuracy.

Feature engineering follows, where domain knowledge is used to create new variables, such as combining aneurysm size with patient smoking status to capture synergistic effects. Features are normalized to ensure equal contribution, similar to standardizing units in a scientific experiment ^(25)^. Traditional methods rarely employed such sophisticated feature creation, relying instead on raw measurements like size or patient age, which overlooked critical interactions. Algorithm selection then determines the best approach for the task. Logistic regression models rupture probability with interpretable coefficients, support vector machines handle nonlinear relationships by mapping data to higher dimensions, random forests combine multiple decision trees to reduce errors in small datasets, and XGBoost refines predictions iteratively for high accuracy ^(26), (27)^. In contrast, traditional statistical models like linear regression were limited to linear relationships and struggled with the complexity of aneurysm data, leading to less accurate predictions ^(24)^.

Model training involves learning patterns from labeled data, such as distinguishing ruptured from unruptured aneurysms, with performance optimized through hyperparameter tuning and evaluated using metrics like area under the curve (AUC) ^(22)^. Traditional approaches often used fixed thresholds or basic risk scores, lacking the adaptability of ML to learn from diverse datasets. Interpretation of ML models is enhanced by tools like Shapley Additive Explanations (SHAP), which quantify each variable’s contribution, providing insights into clinical drivers like aneurysm location or blood pressure ^(28)^. Unlike traditional methods, which offered limited insight into variable importance, ML provides detailed explanations, fostering clinical trust. By capturing nonlinear relationships and integrating diverse inputs, ML models outperform traditional risk scores, offering precise, patient-specific predictions that support decisions like surgical intervention ^(20), (29)^.

### DL: Automated feature extraction

DL, particularly CNNs, automates pattern recognition in raw imaging data, eliminating the need for manual feature selection. These models analyze scans autonomously, identifying aneurysms by learning from large datasets and detecting complex patterns that may be overlooked by human observers ^(30)^. The process begins with image preprocessing, where CTA or MRA scans are resized, normalized, and augmented with transformations like rotation to increase dataset diversity and prevent overfitting. Preserving 3D volumes ensures spatial context is maintained, akin to assembling a complete model from puzzle pieces ^(11)^. Traditional radiology relied on manual inspection by radiologists, who visually scanned images for abnormalities, a process prone to fatigue and variability, often missing subtle aneurysms ^(16)^. DL automates this task, processing entire image volumes with consistent precision.

Model architecture design follows, where CNNs are structured as layered networks. Convolutional layers apply filters to detect features, from simple edges to complex aneurysm shapes, generating feature maps that highlight patterns. Pooling layers reduce computational load by summarizing these maps, while activation functions introduce nonlinearity to capture intricate relationships. Fully connected layers integrate features for tasks like classification or risk prediction ^(31)^. Advanced architectures, such as U-Net for segmentation or vision transformers for 3D analysis, enhance performance by capturing spatial dependencies ^(32), (33)^. Traditional methods lacked such automation, relying on radiologists’ subjective assessments, which were limited to basic visual cues and could not process complex spatial patterns.

Training involves adjusting filter weights using backpropagation to minimize errors, guided by optimization algorithms like Adam. Large datasets are required, often supplemented by transfer learning, where models pre-trained on general images are fine-tuned for aneurysms ^(34)^. In contrast, traditional methods did not leverage large-scale learning, depending on individual expertise. During inference, trained models process new scans, outputting predictions like aneurysm locations or risk probabilities, with post-processing techniques refining results to reduce errors ^(35)^. Explainability is enhanced by tools like Gradient-weighted Class Activation Mapping (Grad-CAM), which generate heatmaps to show decision-driving regions, aligning outputs with clinical needs ^(36)^. Unlike traditional visual assessments, which offer no such transparency, DL provides detailed, automated insights, achieving superior detection and risk prediction accuracy, particularly for complex or small aneurysms ^(11)^.

## AI for Aneurysm Detection

Detecting cerebral aneurysms, especially those smaller than 3 mm or in anatomically complex regions, is crucial yet challenging due to their subtle presentation and variability in radiologist interpretation ^(5)^. AI, particularly DL, automates detection, increasing sensitivity and reducing oversight. Like a high-precision scanner, AI reviews full image volumes from CTA or MRA, standardizing inputs across scanners through preprocessing ^(37)^. This improves on traditional manual review, which is time-consuming and prone to errors, especially with small or obscured aneurysms ^(16)^.

CNNs are also trained on large datasets to detect aneurysmal features, evolving from recognizing basic edges to complex bulges ^(31)^. The model outputs bounding boxes or probability scores highlighting suspected aneurysms. In one study, a CNN identified six sub-3 mm aneurysms missed by three expert radiologists, demonstrating its superior sensitivity ^(38)^. Integrating anatomical constraints, such as vessel-tracking maps, further reduces false positives by cross-referencing predictions with vascular anatomy ^(39)^ which is a step traditional methods lack.

Explainable AI (XAI) tools like Grad-CAM enhance trust by producing heatmaps that highlight AI-detected regions. Semi-supervised learning methods further improve robustness by leveraging unlabeled data ^(36)^. Overall, AI systems now exceed 90% sensitivity and minimize manual effort by combining detection and segmentation, marking a substantial improvement over labor-intensive, variable-prone radiology workflows ^(31), (40)^.

## AI for Aneurysm Rupture Risk Prediction

Accurate rupture risk prediction is vital since most aneurysms remain stable and unnecessary treatment carries risk. Traditional tools like the PHASES score, which considers size, location, and basic clinical risk factors, provide general estimates (AUC ~0.564) but often lack individual precision ^(6), (41)^. Other established benchmarks include radiologist interpretation of CTA/MRA, which remains the standard in current clinical practice but suffers from inter-observer variability and reduced accuracy for small or morphologically complex aneurysms. The ELAPSS score, originally designed to predict aneurysm growth, is also applied as a surrogate for rupture risk but, like PHASES, demonstrates only modest predictive value. Together, these methods form the clinical baselines against which newer AI models must be evaluated.

In contrast, AI enables personalized risk assessment by integrating radiomics, hemodynamic data from computational fluid dynamics simulations (e.g., wall shear stress), and patient information like smoking or hypertension status ^(8), (42), (43)^. ML models―support vector machines, random forests, or XGBoost―can capture complex, nonlinear relationships. DL CNNs analyze imaging directly, while hybrid models combine both for improved performance ^(44), (45)^. Traditional scoring systems like PHASES use fixed weights and cannot account for nuanced interactions. AI models, by contrast, adapt to patient-specific profiles and clinical contexts.

AI-generated risk outputs may include numerical scores or visual risk maps. These tools are validated through cross-validation and balancing methods such as Synthetic Minority Oversampling Technique (SMOTE) to address the rarity of ruptured cases. When compared directly against PHASES, ELAPSS, and radiologist interpretation, several studies report AI-based models achieving AUCs up to 0.864, representing a significant improvement over conventional benchmarks ^(41), (46)^. External validation across institutions further supports their robustness.

Interpretability tools, such as Shapley Additive Explanations (SHAP) and Local Interpretable Model-agnostic Explanations (LIME), help explain how inputs such as wall shear stress or smoking influence model predictions ^(28), (47)^. This transparency is essential for clinical adoption, allowing physicians to trust and act on AI guidance. By offering individualized insights and higher predictive accuracy, AI has the potential to complement existing benchmarks in rupture risk assessment, though rigorous validation remains necessary before clinical adoption.

**Figure 1.**
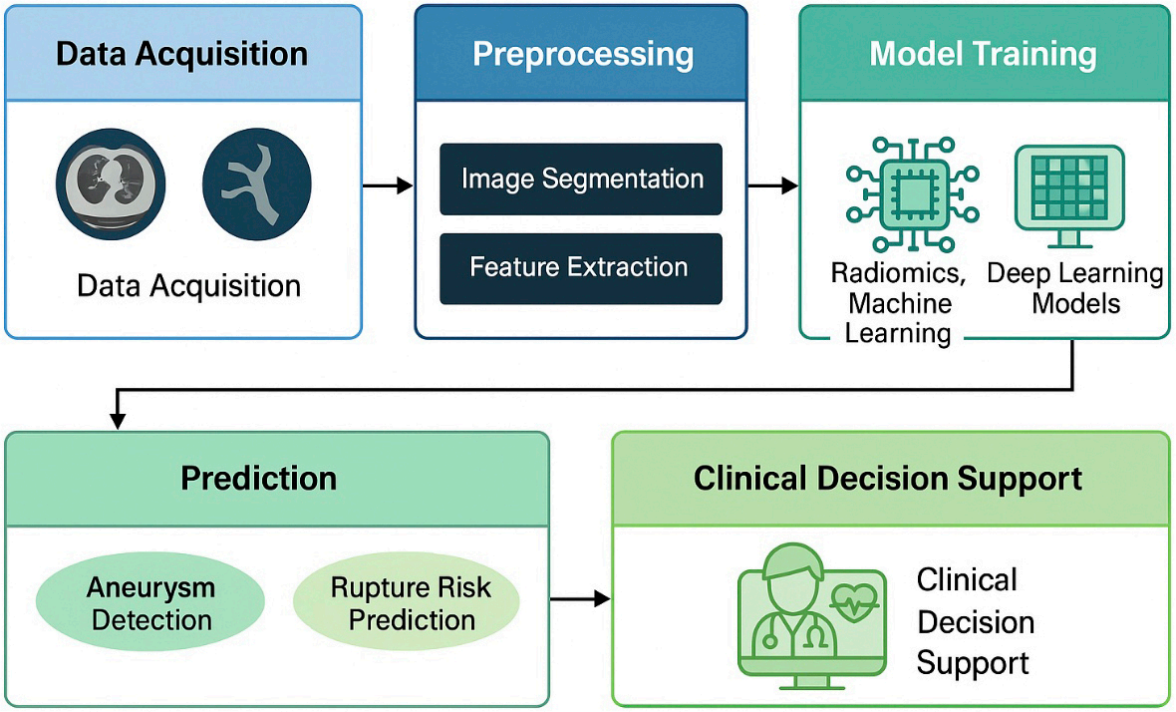
Overview of artificial intelligence applications in cerebral aneurysm imaging. This schematic diagram illustrates the three main artificial intelligence (AI) methodologies (radiomics, machine learning, and deep learning) and their respective roles in cerebral aneurysm detection and rupture risk prediction. Radiomics focuses on quantitative feature extraction from medical imaging; machine learning integrates structured data for risk modeling; and deep learning automates feature recognition directly from imaging inputs. The figure highlights data flow from imaging and clinical sources into AI pipelines, leading to predictive outputs and clinical decision support.

## Challenges and Limitations

Despite their transformative potential, AI methodologies in cerebral aneurysm imaging face significant challenges that hinder clinical adoption. These include technical, methodological, and practical barriers across radiomics, ML, and DL, impacting their effectiveness and integration.

Data scarcity and quality are major hurdles. Cerebral aneurysms, especially ruptured cases, are rare, leading to limited datasets that can cause overfitting, particularly in data-intensive DL models. Variability in imaging modalities, resolutions, and contrast agents across institutions further degrades data quality, affecting model performance ^(11), (48)^. Traditional methods relied on manual interpretation, less sensitive to dataset size but prone to human error. Solutions like transfer learning, semi-supervised learning, and synthetic data generation can expand effective dataset sizes ^(34)^.

Moreover, generalizability is also a critical challenge. Models trained on single-institution data often fail with new scanners, protocols, or patient populations, limiting real-world applicability. Radiomics is sensitive to segmentation variability, while ML and DL struggle with domain shifts from differing imaging hardware ^(48), (49)^. Traditional methods, although less generalizable due to subjective interpretation, avoided these technical issues. Domain adaptation, external validation, and federated learning can enhance model robustness ^(50)^.

Interpretability and trust pose significant barriers, especially for DL’s “black-box” nature, which obscures decision-making and reduces clinician confidence in high-stakes settings. ML models also require transparency tools ^(11)^. XAI methods like Grad-CAM and SHAP improve clarity but need standardization for clinical use ^(28), (36)^. Traditional methods were interpretable but relied on subjective judgment. Interactive interfaces and clinical rule integration can bridge this trust gap ^(51)^.

Additionally, standardization issues hinder reproducibility. Radiomics features vary across software and protocols, and ML and DL models lack uniform architectures or evaluation metrics, complicating comparisons ^(13), (23)^. Traditional methods faced inconsistent diagnostic criteria but were less dependent on technical standardization. Initiatives such as the Image Biomarker Standardization Initiative and the Transparent Reporting of a multivariable prediction model for Individual Prognosis Or Diagnosis - Artificial Intelligence (TRIPOD-AI) guidelines aim to address these issues ^(13), (52)^.

False positives and workflow integration present practical challenges. Detection models may misidentify normal structures as aneurysms, increasing radiologist workload and patient anxiety ^(37)^. Integration with electronic health records or imaging systems is often lacking, disrupting workflows. Traditional methods avoided false positives through human judgment but were slower. Anatomical priors and user-centered design for decision support systems can mitigate these issues ^(39), (53)^.

Next, ethical and regulatory considerations are critical. Bias in training data risks inequitable outcomes, and regulatory frameworks like the United States Food and Drug Administration’s (FDA) ‘Software as a Medical Device’ demand rigorous validation ^(51), (53)^. Traditional methods faced fewer regulatory hurdles but lacked precision. Diverse datasets and SPIRIT-AI guidelines ensure fairness ^(54)^.

Computational and resource barriers also limit accessibility. DL requires significant computational power, while radiomics and ML demand specialized software and expertise ^(18)^. Implementation costs challenge low-resource settings. Traditional methods were resource-light but limited by human effort. Cloud-based platforms and lightweight models offer scalable solutions ^(50)^. Addressing these challenges through collaboration, standardized protocols, and intuitive designs will enable AI to surpass traditional approaches in aneurysm management.

Finally, rigorous clinical validation remains a critical barrier to adoption. Most existing AI models are trained and tested retrospectively on single-institution datasets, which risks overfitting and inflates apparent performance. For AI to be credibly positioned as superior or complementary to existing approaches, prospective, multi-center clinical trials and external test sets are essential. Moreover, models must be benchmarked directly against current standards―radiologist interpretation, PHASES, and ELAPSS―to demonstrate true incremental value. Without such systematic validation, claims of superiority remain preliminary and cannot yet support widespread clinical implementation.

## Future Directions and Innovation

### Integrating diverse data sources for comprehensive risk assessment

The future of AI in cerebral aneurysm care lies in its capacity to integrate multiple data sources, creating detailed, patient-specific risk profiles that surpass the limitations of current imaging-focused models. Contemporary approaches often rely on radiomic features or basic clinical variables, such as aneurysm diameter or patient age, which fail to capture the multifaceted interplay of hemodynamic, biological, and lifestyle factors influencing aneurysm behavior. Integrating diverse data involves combining high-resolution imaging from CTA or MRA with clinical records, including hypertension or smoking history, laboratory biomarkers such as C-reactive protein for inflammation or D-dimer for coagulopathy, and genetic profiles identifying vascular vulnerabilities. Additionally, real-time physiological data from wearable devices, such as blood pressure monitors or smartwatches tracking heart rate variability, can detect dynamic stressors, such as nocturnal hypertension, that may exacerbate aneurysm instability. This comprehensive approach constructs a multidimensional patient profile, offering superior predictive accuracy compared to traditional tools like the PHASES score, which relies on generalized population-based metrics ^(24)^. Advanced computational frameworks, such as graph neural networks and transformers, are well-suited for this task, as they adeptly model complex relationships across heterogeneous data. Graph neural networks represent patients as interconnected nodes, linking imaging characteristics to genetic or clinical factors, while transformers employ attention mechanisms to prioritize critical interactions, such as wall shear stress influencing inflammatory pathways ^(50)^. Radiogenomics, an emerging field, further enhances this approach by correlating imaging phenotypes with molecular profiles, identifying aneurysm subtypes with distinct rupture risks or therapeutic responses. For instance, aneurysms exhibiting irregular morphology on CTA may be associated with upregulated matrix metalloproteinase expression, indicating wall instability amenable to targeted pharmacological interventions ^(45)^. Challenges include data heterogeneity across institutions and the computational demands of processing multimodal inputs. Standardizing data formats, employing transfer learning to accommodate variability, and leveraging cloud-based infrastructure can mitigate these issues, drawing on successful models like the Human Heredity and Health in Africa initiative, which harmonized genomic and clinical data across diverse populations ^(55)^. By synthesizing these diverse data sources, AI can facilitate personalized treatment and follow-up strategies, advancing the paradigm of precision medicine in aneurysm management.

### Enhancing model transparency for clinical trust

The opacity of DL models, often characterized as inscrutable, poses a significant barrier to their adoption in high-stakes neurovascular care, where clinicians require clear rationales to trust AI recommendations. Enhancing model transparency through XAI is essential to foster a collaborative partnership between AI systems and healthcare professionals. Techniques, such as SHAP, quantify the contribution of individual features, such as aneurysm aspect ratio or wall shear stress, to risk predictions, while Grad-CAM visually highlights critical regions on imaging scans, enabling clinicians to validate AI outputs against their clinical judgment ^(28), (36)^. For example, if an AI model identifies a high-risk aneurysm, SHAP may indicate that irregular wall morphology and elevated blood pressure are primary drivers, allowing clinicians to contextualize the prediction within their expertise. Incorporating physiological principles into model architectures further enhances credibility by ensuring outputs align with established medical knowledge, such as the role of high wall shear stress in aneurysm wall degradation ^(9)^. Hybrid models, combining ML with rule-based logic, can emulate clinical decision-making processes, recommending interventions for aneurysms with specific risk factors, such as irregular morphology or concurrent hypertension, thereby aligning with clinicians’ reasoning. Interactive XAI systems enable clinicians to explore hypothetical scenarios, such as the impact of initiating antihypertensive therapy or smoking cessation on rupture risk, supporting shared decision-making with patients ^(51)^. Natural language processing interfaces can translate complex model outputs into concise, clinically relevant summaries, reducing cognitive burden in high-pressure environments. Establishing standardized metrics for explanation reliability, such as fidelity and stability, is critical to ensure consistent interpretability. Prospective studies must evaluate whether these tools reduce diagnostic errors or improve patient outcomes, addressing usability concerns. Collaborative efforts, such as those led by the XAI in Healthcare consortium, can establish best practices, ensuring XAI systems are both trustworthy and practical for clinical adoption ^(56)^. By prioritizing transparency, these advancements position AI as a reliable ally in aneurysm care.

### Conducting rigorous clinical trials for real-world validation

The reliance on retrospective datasets limits the generalizability of current AI models, necessitating rigorous prospective clinical trials to validate their performance in real-world settings. Unlike retrospective analyses, which risk overfitting to historical data, prospective trials evaluate AI tools across diverse patient populations and clinical environments, assessing their impact on critical outcomes, such as reduced rupture rates, fewer unnecessary interventions, or enhanced diagnostic efficiency. A multicenter trial, for instance, could compare AI-assisted risk stratification to traditional tools like the PHASES score, measuring metrics such as sensitivity, specificity, and cost-effectiveness ^(53)^. These trials should also investigate whether AI augments clinical decision-making, identifying high-risk aneurysms overlooked by conventional methods or reducing the burden of unwarranted procedures. To confirm true incremental value, prospective studies must explicitly compare AI performance against established clinical benchmarks, including radiologist interpretation, PHASES, and ELAPSS. Regulatory frameworks, such as the FDA’s ‘Software as a Medical Device’ guidelines, mandate that AI tools demonstrate safety, efficacy, and adaptability to evolving data distributions ^(57)^. Post-market surveillance through registries can monitor long-term performance, detecting issues such as model drift or biases in specific demographics. Guidelines like SPIRIT-AI and CONSORT-AI ensure transparent trial design and reporting, addressing data sources, model updates, and ethical considerations ^(54)^. International collaboration, facilitated by consortia such as the International Consortium for Intracranial Aneurysm Studies, can harmonize trial protocols, ensuring models perform robustly across diverse populations and imaging protocols ^(1)^. Financial and infrastructural barriers may impede trial implementation, particularly in resource-constrained settings. Public-private partnerships and cloud-based trial platforms can alleviate these challenges, while regulatory sandboxes enable controlled testing before widespread deployment. Through rigorous clinical validation, these trials will provide the evidence needed for regulatory approval and clinical adoption, ensuring AI delivers measurable benefits to patients. Ultimately, validation should not only demonstrate statistical accuracy but also show clinical utility beyond conventional standards. Only if AI consistently outperforms or complements established benchmarks such as PHASES, ELAPSS, and radiologist interpretation can it justify integration into routine practice.

### Enabling secure data collaboration through federated learning

Limited data availability, compounded by the rarity of aneurysm cases and stringent privacy regulations such as the General Data Protection Regulation (GDPR) and the Health Insurance Portability and Accountability Act (HIPAA), continues to hinder large-scale data sharing in medical AI research. Federated learning offers a transformative solution by enabling collaborative model development without compromising patient confidentiality. In this approach, institutions train local models on their proprietary datasets and share only encrypted model parameters, such as neural network weights, with a central server that aggregates them into a global model ^(48)^. This method allows AI to leverage diverse datasets from institutions worldwide, enhancing generalizability across different imaging modalities, protocols, and patient demographics. For example, a federated model could incorporate CTA scans from North America, Europe, and Asia, capturing variations in aneurysm morphology and risk profiles. Differential privacy techniques, which introduce controlled noise to model updates, and secure multi-party computation further safeguard data, ensuring compliance with regulatory standards ^(23)^. Challenges include data heterogeneity, such as variations in imaging parameters or annotation practices, which can compromise model performance. Harmonization protocols, such as those established by the Image Biomarker Standardization Initiative, ensure consistent feature extraction and labeling ^(13)^. Lightweight federated learning approaches, such as federated distillation, reduce computational requirements, enabling participation from resource-limited institutions ^(58)^. Successful applications in oncology, such as the Federated Tumor Segmentation initiative, demonstrate the potential to enhance model robustness and reduce bias, providing a model for aneurysm research ^(59)^. By facilitating secure, global collaboration, federated learning accelerates the development of equitable, high-performing AI models for aneurysm care.

### Developing intuitive clinical tools for seamless integration

To realize AI’s potential in aneurysm management, it must be seamlessly integrated into clinical workflows through intuitive Clinical Decision Support Systems (CDSS). These systems bridge the gap between AI models and clinical practice by interfacing with electronic health records and imaging platforms, delivering real-time, context-aware recommendations. For instance, a CDSS could flag high-risk aneurysms during scan reviews or track morphological changes over time, supporting longitudinal patient management. Reinforcement learning enables these systems to adapt based on clinician feedback, refining recommendations as physicians adjust predictions to account for patient-specific factors, such as a family history of rupture ^(60)^. User-centered design is crucial, with intuitive interfaces displaying risk scores, imaging annotations, and treatment suggestions tailored to clinical needs. Natural language processing can distill complex outputs into actionable insights, such as recommending follow-up imaging for stable aneurysms, minimizing cognitive burden. Compatibility with existing systems, such as Epic Systems Corporation (Epic) or Picture Archiving and Communication Systems (PACS), ensures seamless integration, while cloud-based platforms enhance scalability across diverse healthcare settings ^(53)^. Comprehensive training programs, including simulation-based learning, build clinician confidence, while stakeholder engagement during system design fosters adoption ^(61)^. Ethical considerations include preventing over-reliance on AI, maintaining clinician autonomy, and transparently documenting limitations. Patient engagement tools, such as shared decision-making modules, align with ethical guidelines like SPIRIT-AI, empowering patients in treatment planning ^(54)^. By embedding AI into intuitive, adaptive systems, CDSS transforms it into a trusted clinical partner, enhancing decision-making and patient outcomes.

## Conclusion

AI is a vital tool in cerebral aneurysm imaging, enhancing detection, characterization, and risk prediction beyond traditional methods. Radiomics, ML, and DL extract complex features, model individualized risk, and automate interpretation. Multimodal fusion, XAI, clinical trials, and federated learning promise proactive, personalized care, blending human expertise with AI for better outcomes. However, meaningful clinical adoption will depend on rigorous validation against established benchmarks such as radiologist interpretation, PHASES, and ELAPSS. Demonstrating not only statistical accuracy but also clear clinical utility beyond these standards is essential. Only through such benchmark-based, prospective validation can AI justify integration into routine cerebrovascular practice.

## Article Information

### Author Contributions

Conceptualization, methodology, writing - original draft, review & editing: Duong Le.

### Conflicts of Interest

None
